# Case report: Diagnostic trap: a extremely rare metastatic myoepithelial carcinoma of breast

**DOI:** 10.3389/fonc.2024.1436178

**Published:** 2024-10-16

**Authors:** Shuai Luo, Xiaoxue Tian, Ting Xu, Jinjing Wang

**Affiliations:** Department of Pathology, Affiliated Hospital of Zunyi Medical University, Zunyi, Guizhou, China

**Keywords:** parotid gland, myoepithelial carcinoma, mammary gland, pathological diagnosis, metastasis

## Abstract

**Background:**

Myoepithelial carcinoma (MECA) is a malignant tumor primarily affecting the salivary gland, most frequently in the parotid gland. It can manifest as primary or secondary to pleomorphic adenoma or benign myoepithelioma. MECA exhibits aggressive behaviors. In particular, primary MECA is more aggressive, frequently recurring or metastasizing distantly. Its morphological and immunohistochemical characteristics overlap with various tumors, posing challenges in its recognization as a distinct entity. Consequently, MECA may be frequently misdiagnosed, mainly when occurred in the mammary gland. This chance for misdiagnosis poses significant challenges in clinical diagnosis and treatment.

**Case demonstration:**

A 77-year-old woman with a history of pleomorphic adenoma presented with a palpable lump in the right breast for 3 months. Subsequent core needle biopsy (CNB) and modified radical mastectomy were performed, with samples subjected to histopathological examination. Based on the patient’s history, histomorphologic features, immunohistochemistry (IHC) results and results of FISH, the pathological diagnosis confirmed MECA in the mammary gland. Postoperative chemotherapy was administered, and the patient exhibited a favorable prognosis during a 40-month follow-up period.

**Conclusions:**

Primary MECA in the mammary gland is exceedingly rare, metastasis from the salivary gland MECA to the mammary gland is even rarer and has not been previously reported. This study presents the first documented case of MECA originating from the parotid gland metastasizing to the mammary gland (also known as breast). Highlighting this case aims to raise awareness among clinical pathologists to prevent underdiagnosis and misdiagnosis of this tumor entity.

## Introduction

Myoepithelial carcinoma (MECA) is an uncommon epithelial ovarian tumor primarily found in salivary glands, representing approximately 0.2% of all salivary gland tumors (1). Histologically, MECA is characterized by its predominant composition of myoepithelial cell (MEC) and aggressive growth behavior (2). The diagnostic criteria for MECA remain unclear, although Stromeyer et al. initially described this solid tumor in 1975 (3). The structure of MECA typically exhibits a multinodular or sheet-like growth pattern or both against a background of mucinous or collagenous stroma. Cytologically, it may present as epithelioid, plasma cell-like, spindle cell-like, clear cell-like, or a mixture of these cell types (4). MECA’s histological feature is its morphologic heterogeneity, characterized by different cell types and growth patterns. This heterogeneity has previously led to misdiagnoses as various salivary gland cancers or even classification as a “malignant mixed tumor”. Immunohistochemical (IHC) testing requires cytokeratin (CK) positivity (100%) and expression of at least one myoepithelial marker for MECA diagnosis (5). MECA is molecularly characterized by transforming growth factor beta receptor 3-pleomorphic adenoma gene 1 (TGFBR3-PLAG1) and fibroblast growth factor receptor 1-pleomorphic adenoma gene 1 (FGFR1-PLAG1) gene fusions (6) as well as EWSR1 gene rearrangements (7).In this case, the PLAG1(8q12) break/FISH analysis showed a PLAG1 gene break.

## Case demonstration

**Figure 1:**
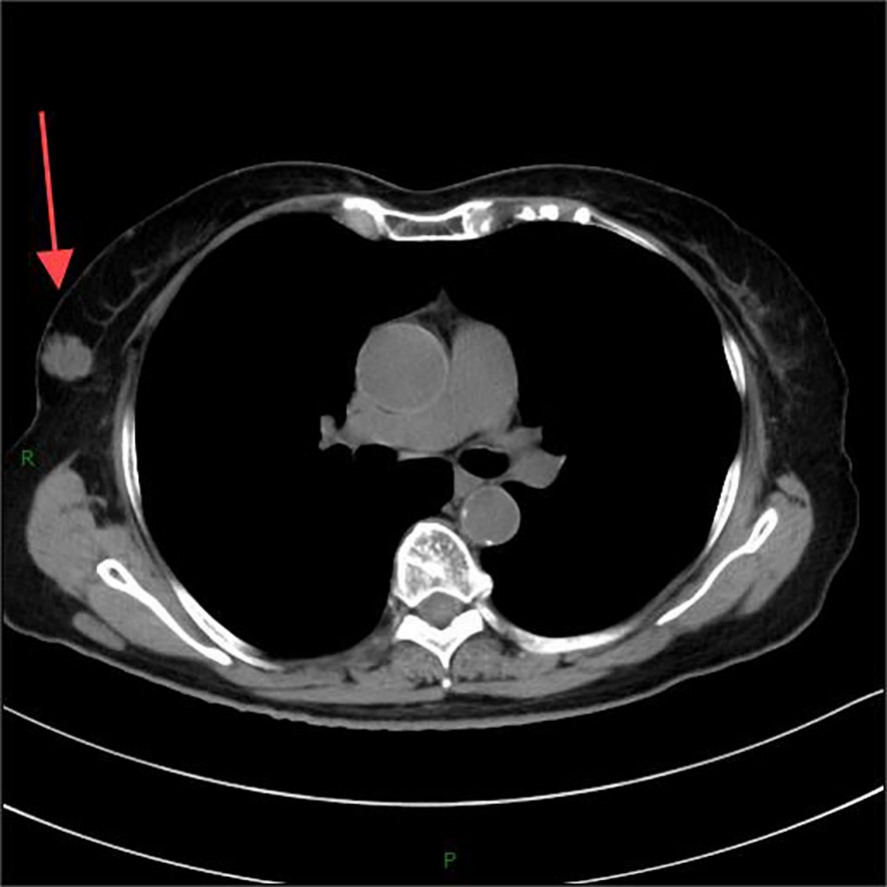
Computed tomography (CT) imaging confirmed the presence of a lobulated mass in the upper outer quadrant of the right breast.

A 77-year-old woman was admitted to the hospital due to a lump discovered in her right breast during a physical examination 3 months prior. Physical examination revealed symmetrical breasts without inversion of the nipples, skin scarring, redness, swelling, ulcers, Peau d’orange, or dimpling. A palpable mass measuring approximately 31 mm×20 mm was identified below the areola in the 10 o’clock direction of the right breast; it was firm to touch, exhibited fair mobility, and did not elicit compression pain. Computed tomography (CT) imaging confirmed the presence of a lobulated mass in the upper outer quadrant of the right breast(**Figure 1**), exhibiting internal septations and clear margins, approximately 23 mm×19 mm×18 mm in size, classified as Breast Imaging Reporting and Data System (BI-RADS) category 6.

A core needle biopsy (CNB) was subsequently performed. Microscopically, the tumor cells exhibited a spindle-shaped arrangement, forming abundant, solid sheets comprising round, ovoid, polygonal, spindle, and signet-ring cells with eosinophilic cytoplasm, prominent nucleoli, and a mucus-rich interstitium. Mitotic count was not easily discernible. IHC results indicated negative estrogen receptor (ER) and progesterone receptor (PR) status, Human Epidermal Growth Factor Receptor 2 (HER-2) negativity, positive E-cadherin, cytoplasmic positivity for P120, and positive GATA binding protein 3 (GATA3), vimentin, and Ki-67 (10%+) expression in tumor cells. Pathological diagnosis following CNB revealed invasive ductal carcinoma of the breast (non-specific type), histologic grade 2.

Neoadjuvant chemotherapy was initiated after that. Follow-up breast ultrasound imaging conducted 3 months post-chemotherapy did not show significant tumor shrinkage. Subsequently, a modified radical mastectomy was performed. Gross examination revealed a spindle-shaped skin breast radical specimen measuring 20 mm×17 mm×2 mm in size, with a 13 mm×7 mm spindle-shaped skin area, a 1 cm diameter nipple, and a 2 mm×2 mm×1 mm grayish solid mass located 7 mm from the nipple, moderately differentiated and poorly defined from the surrounding tissue.

**Figure 2:**
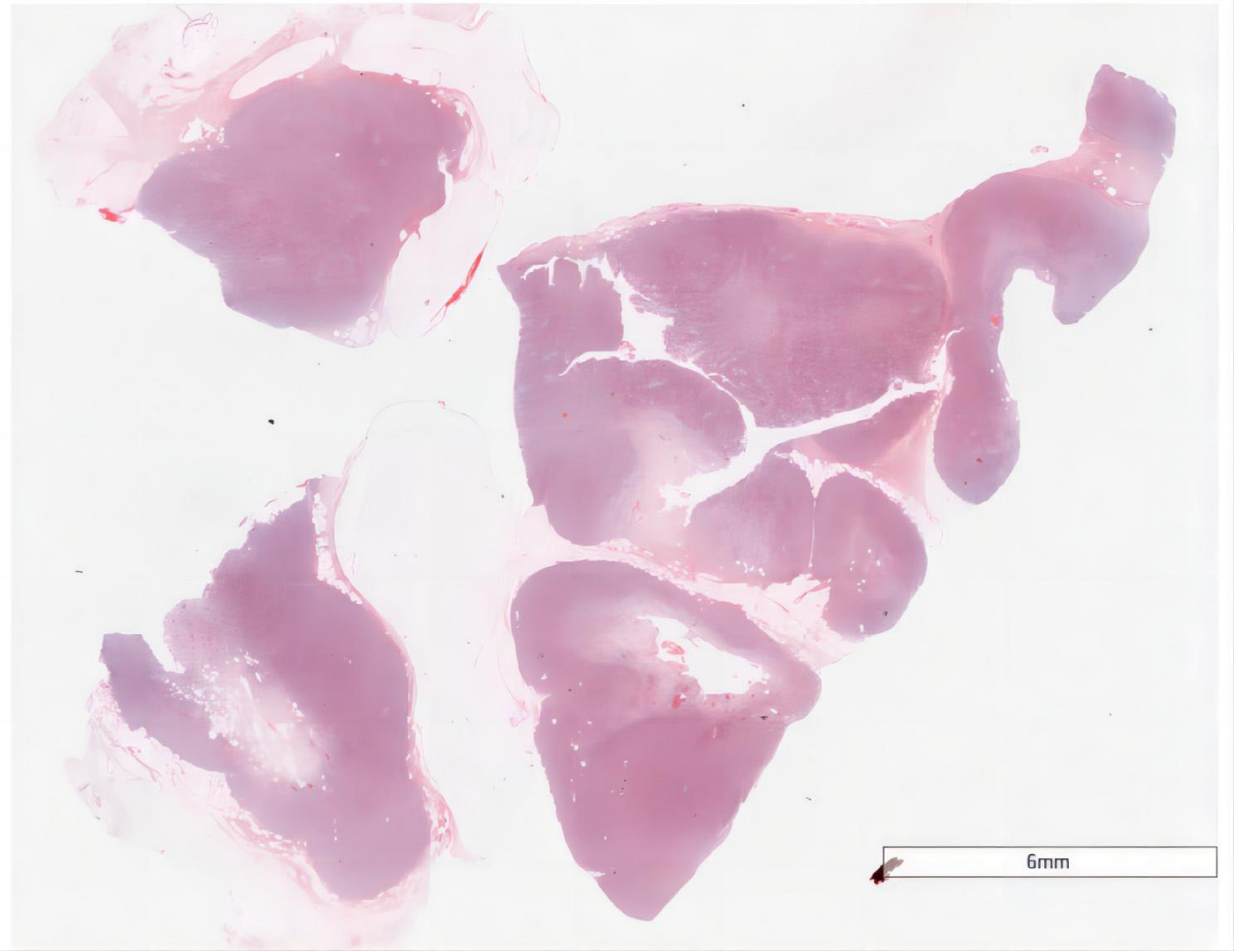
Low-magnification imaging depicted a nodular tumor that remained well-demarcated from the surrounding normal breast tissue, exhibiting expansive growth without a distinct intact fibrous capsule.

**Figure 3:**
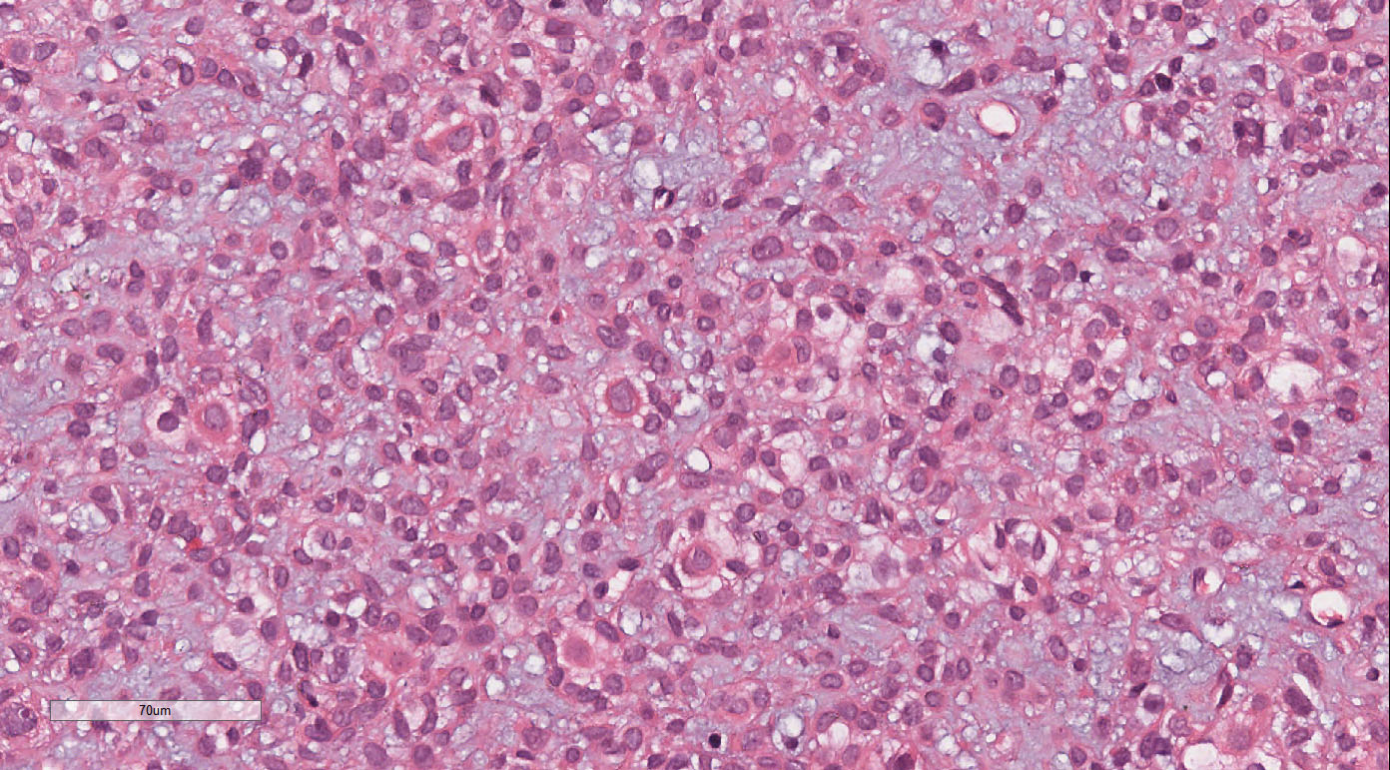
High-magnification imaging revealed abundant solid sheets of round, ovoid, polygonal, spindle, and signet-ring cells. These cells exhibited eosinophilic cytoplasm, prominent nucleoli, a mucus-rich interstitium, and a mitotic count of approximately 1–3 per high-power field.

**Figure 4:**
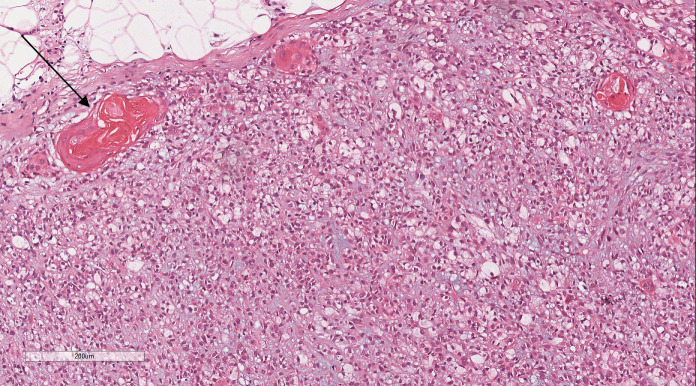
Within the tumor, focal areas of squamous metaplasia (black arrow).

**Figure 5:**
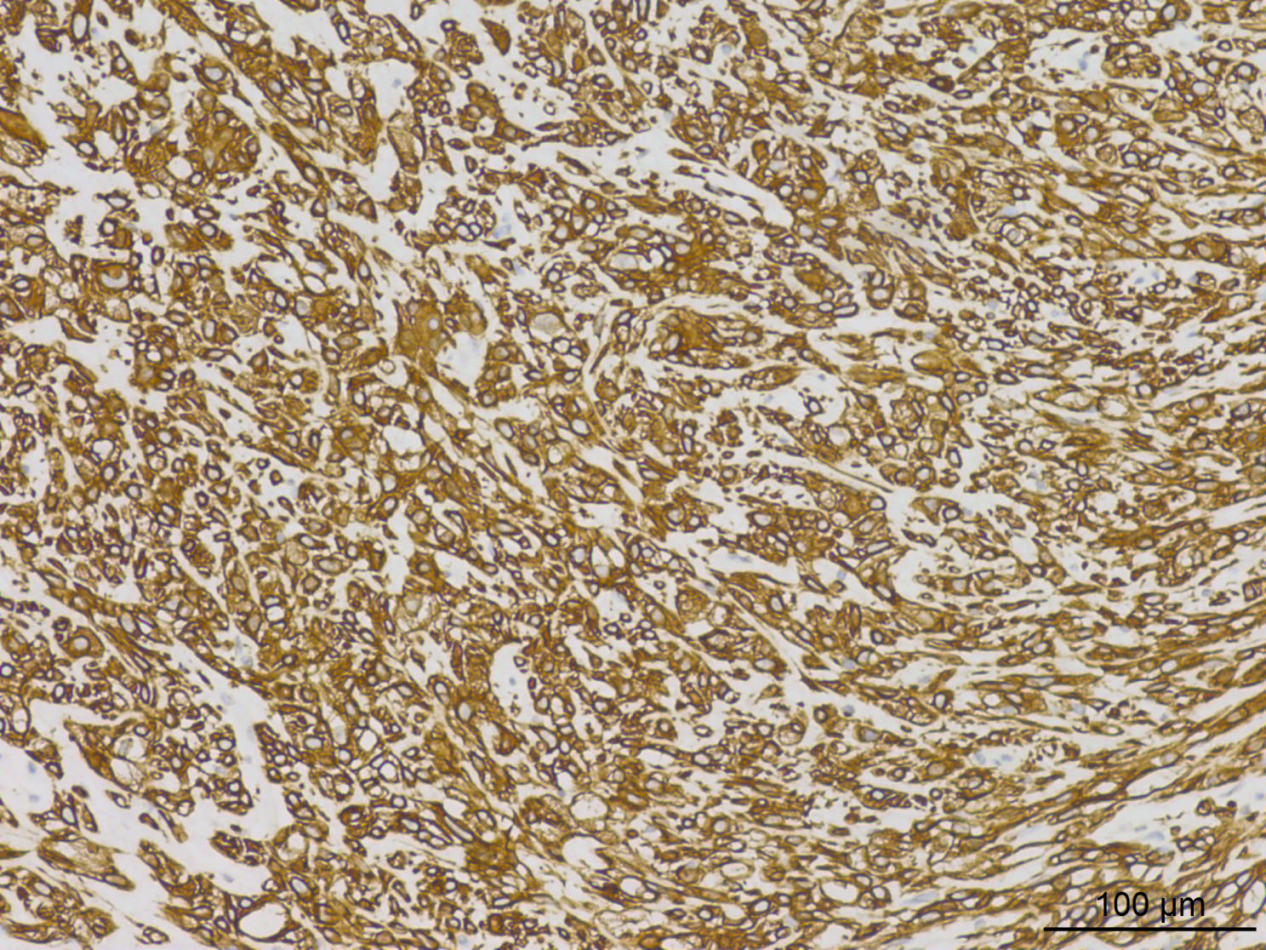
IHC results indicated positive expression of CK5/6 in tumor cells.

**Figure 6:**
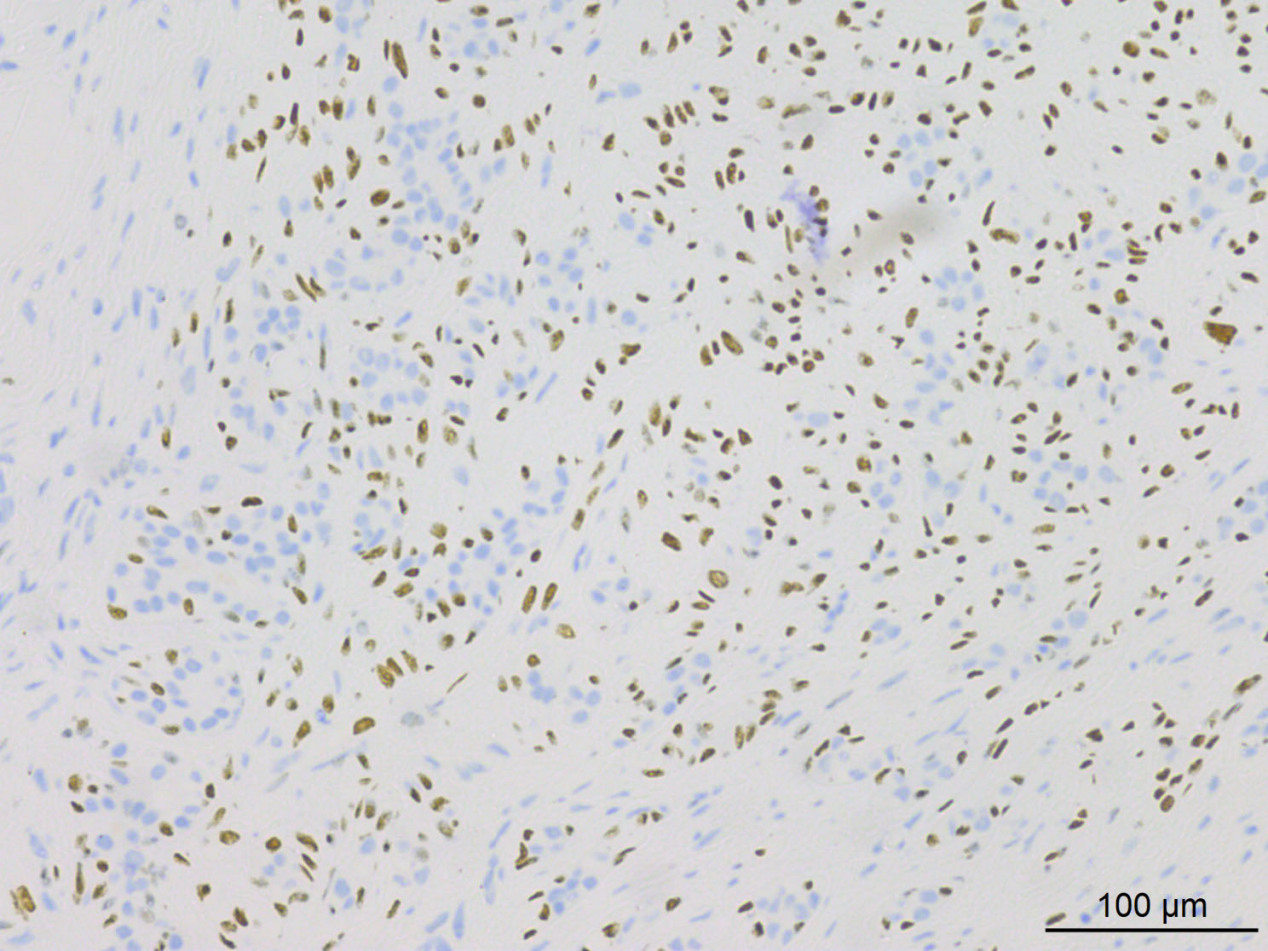
IHC results indicated positive expression of P63 in tumor cells.

**Figure 7:**
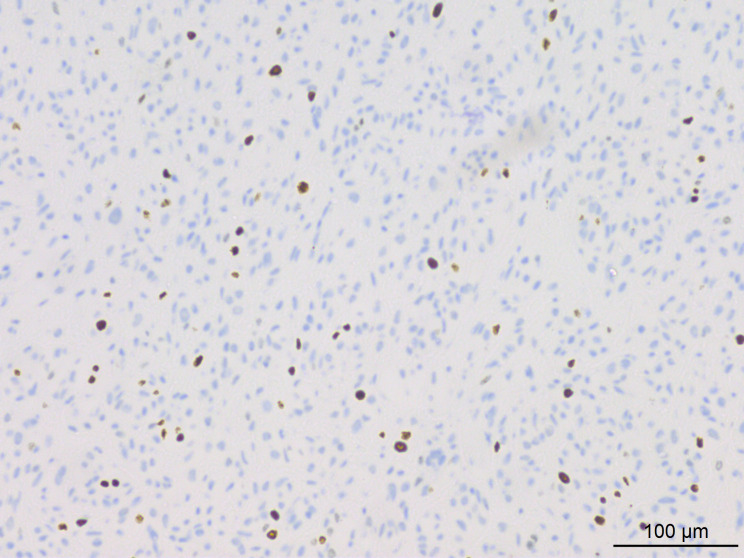
IHC results indicated approximately 10% of tumor cells exhibited positive staining for Ki-67.

Histopathological analysis at low magnification (**Figure 2**) revealed a nodular tumor, clearly demarcated from the adjacent normal breast tissue, exhibiting expansive growth without an intact fibrous envelope. High-magnification imaging (**Figures 3**, **4**) revealed abundant, solid sheets of cells with various morphologies, consistent with the earlier description, and a mitotic count averaging 1–3 per high-power field (HPF). Squamous metaplasia and pseudocysts were observed focally within the tumor, along with small foci of foam histiocytes, and tumor thrombosis was detected in the vasculature. IHC revealed positive staining for vimentin, GATA3, E-cadherin, p-120 (cytoplasmic), cytokeratin, CK5/6 (**Figure 5**), P63 (**Figure 6**), P40, S-100, CK7 and CD117 in the ductal epithelium, weak staining for smooth muscle actin (SMA), and negative staining for ER, PR, and HER-2, and a 10% proportion of staining for Ki-67 (**Figure 7**).

**Figure 8:**
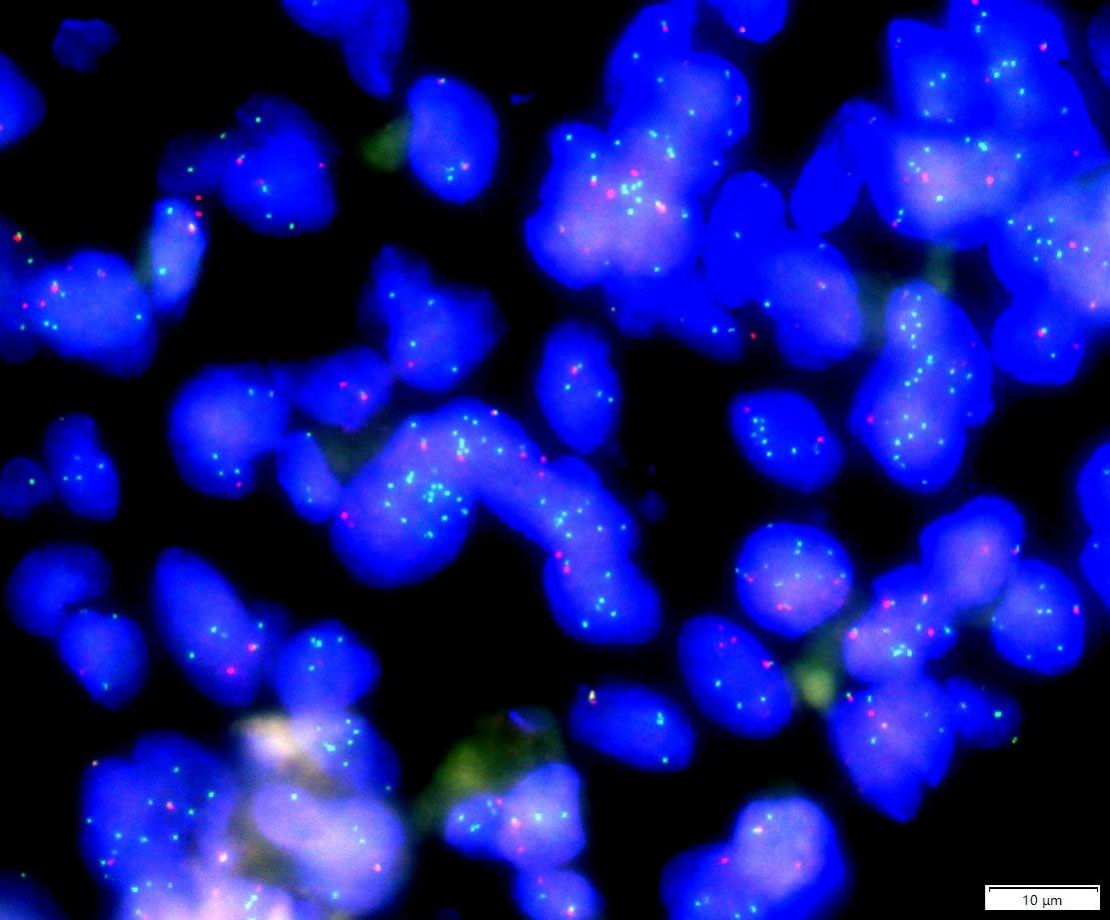
PLAG1(8q12) break/FISH results showed PLAG1 gene break, and the proportion of cells with green and red signal break was about 63% (63/100). (The red signal R and the green signal G represent the two ends of the PLAG1 break point, respectively, and F represents the red and green signals are not separated.).

Fluorescence *in situ* hybridization (FISH) for PLAG1(8q12) break-apart was performed on tumor cells (**Figure 8**), which revealed PLAG1 gene break-apart in 63% of the tumor cells (break-apart rate was greater than the 15% threshold).

Based on the histomorphological features, IHC results and results of FISH, the initial diagnosis was MECA in the right mammary gland. Considering the patient’s medical history, which included surgery for a pleomorphic adenoma on the right side 19 years ago and recurrence of an ipsilateral parotid gland tumor 5 years ago, morphological and IHC findings from other affected sites (right face, right clavicle, and right ribs) were concordant with the parotid gland. Consequently, the patient was pathologically diagnosed with multiple metastases of MECA.

In this case, the final diagnosis of MECA in the right mammary gland was established through a physical examination, which revealed a right breast mass, corroborated by the patient’s medical history, histomorphological features, and IHC results.

Following surgical treatment and chemotherapy, the patient showed a favorable prognosis during the 40-month follow-up period.

## Discussion

MECA is a rare tumor in the salivary glands, included in the second edition of the World Health Organization’s Histological Classification of Salivary Gland Tumors since 1991 (8).

It may arise *de novo* or in association with pleomorphic adenoma (MECA ex pleomorphic adenoma) (5). While primary MECA is generally considered more aggressive (9), our reported case from a pleomorphic adenoma exhibits similar aggressiveness.

Formerly known as malignant mixed tumor or malignant myoepithelioma, MECA may involve major or minor salivary glands, with the parotid gland being the most common site, followed by the palate and submaxillary glands (10). It typically manifests as a well-defined mass devoid of an envelope, appearing grayish or grayish-yellow upon sectioning and presenting a firm consistency.

Histologically, MECA is characterized by an infiltrative growth pattern comprising MECs (8). This tumor exhibits a diverse array of cell types and structural arrangements, the exact histological characteristics of which remain to be fully elucidated (1). Our reported case aligns histologically with existing literature, displaying various cytologic types, including round, ovoid, polygonal, spindle, and signet-ring cells. According to the literature, the histological classification of MECA is predicated on the predominant type present, categorized into epithelioid, clear cell, plasma cell, and spindle cell subtypes. Cases presenting with a multitude of two or more cell types are classified as mixed subtypes (11–13). Accordingly, the case described herein falls under the mixed subtype classification.

At the IHC test, a positive test for CK (100%) and expression of at least one myoepithelial marker such as S-100, SMA, calponin, myosin, SRY-Box Transcription Factor 10 (SOX10), Glial Fibrillary Acidic Protein (GFAP), P40, or P63 are typically required to diagnose MECA (5). The IHC findings in this case are consistent with those reported in the literature. Generally, MECA presents histological challenges, exhibiting different morphological features and heterogeneous IHC results.

MECA tends to be locally invasive with varying clinical outcomes. Local or distant metastasis typically occurs, with the lungs being the most frequent site of metastasis, followed by bone, skin, liver, brain, and other sites. This case, metastasizing to the breast, represents the first reported instance of MECA in the mammary gland. Evidence suggests that cytologically mild MECA (characterized by deficient mitotic activity) may recur and result in mortality (13–15). However, tumors displaying aggressive histologic features (such as cytologic abnormalities, increased mitotic activity, and necrosis) occasionally exhibit relative inertness (13, 16, 17). The case described herein aligns with these findings, showcasing mild cytology, rare nuclear divisions, absence of necrosis, local recurrence, multiple distant metastases, and aggressiveness. However, vascular invasion was present in this case, occurring within a pleomorphic adenoma. Tumor necrosis, occurrence within a pleomorphic adenoma, and vascular invasion have been significantly associated with disease-free survival (18). Despite its lengthy course, recurrence, multiple metastases, and high invasiveness, the patient survived, underscoring the low-grade malignant nature of MECA.

Regarding treatment, surgical intervention is the primary approach for managing MECA, while the efficacy of radiotherapy and chemotherapy remains uncertain (19).

MECA warrants differential diagnosis from several tumors. (1) Differentiation from myoepithelioma: while myoepithelioma shares cellular and IHC similarities with MECA, MECA lacks infiltrative growth, and its cellular heterogeneity is less pronounced; IHC staining with the Ki-67 antibody, characterizing cellular proliferative activity, can aid in distinguishing benign and malignant myoepithelioma, with MECA diagnosed when the Ki-67 index exceeds 10% (20). (2) Differentiation from pleomorphic adenoma: Pleomorphic adenoma features a dual-layered structure comprising epithelial and myoepithelial components and various cellular elements, including mucinous and cartilaginous mesenchyme. (3) Differentiation from pleomorphic adenocarcinoma: Both tumors may exhibit mucus-like mesenchyme and share certain cell types with similar IHC features, although pleomorphic adenocarcinoma presents histological features typified by a target-like arrangement of tumor cells around neurovascular structures. (4) Differentiation from epithelial-myoepithelial carcinoma (EMC): EMCs typically exhibit a characteristic cellular arrangement, with conduit cells encircled by multiple layers of MECs, typically with transparent cytoplasm (5), while this arrangement is absent in MECA. (5) Differentiation from mucoepidermoid carcinoma: Mucoepidermoid carcinoma comprises epidermal, mesenchymal, and mucus-like cells (21). While mucoepidermoid carcinoma tests negative for S-100 and myoepithelial markers, MECA demonstrates positive expressions for S-100 and MECs. Furthermore, mucoepidermoid carcinoma involves a MAML2 gene fusion (22), whereas MECA is associated with a PLAG1 gene rearrangement. (6) Differentiation from solid adenoid cystic carcinoma (solid ACC): Solid ACC exhibits significant cellular heterogeneity, numerous nuclear divisions, cells displaying basal-like characteristics, a high histologic grade, and MYB-NFIB fusion molecules (23).

Given its occurrence in the mammary gland, the tumor must be distinguished from rarer breast tumors.

1. Differentiation from metaplastic carcinoma: The morphology and IHC results of metaplastic carcinoma overlap with those of MECA; however, differentiation is possible through molecular genetics. The molecular pathology of MECA is characterized by PLAG1 fusion, whereas metaplastic carcinoma involves TP53 mutation (24).

2. Differentiation from secretory carcinoma of the breast (SCB): SCB typically exhibits distinctive pathohistological features, including abundant eosinophilic secretions inside and outside tumor cells, resembling thyroid follicles, and even absorptive vacuoles. IHC testing of SCB shows negativity for p63, calponin, CK14, SMA, and CK5/6, with an ETV6-NTRK3 gene fusion (25).

## Conclusion

MECA presents diagnostic challenges due to its heterogeneous cytology and IHC profiles. Typical MECA structures include multinodular or sheet-like growth patterns or both. Cytologically, MECA manifests as a neoplastic proliferation of MECs with varying morphologies. Despite its mild morphological features, MECA demands vigilance due to its potential for aggressive behavior, and distant and local recurrence risk.

## Data Availability

The original contributions presented in the study are included in the article/Supplementary Material. Further inquiries can be directed to the corresponding author.
